# Supplementary material for: A DOT1B/Ribonuclease H2 Protein Complex Is Involved in R-Loop Processing, Genomic Integrity, and Antigenic Variation in Trypanosoma brucei
Source: mBio. 2021 Nov 9;12(6):e01352-21. doi: 10.1128/mBio.01352-21 (PMC8576533; doi:10.1128/mBio.01352-21)
Supplement: FIG S1 [file mbio.01352-21-sf001.pdf]

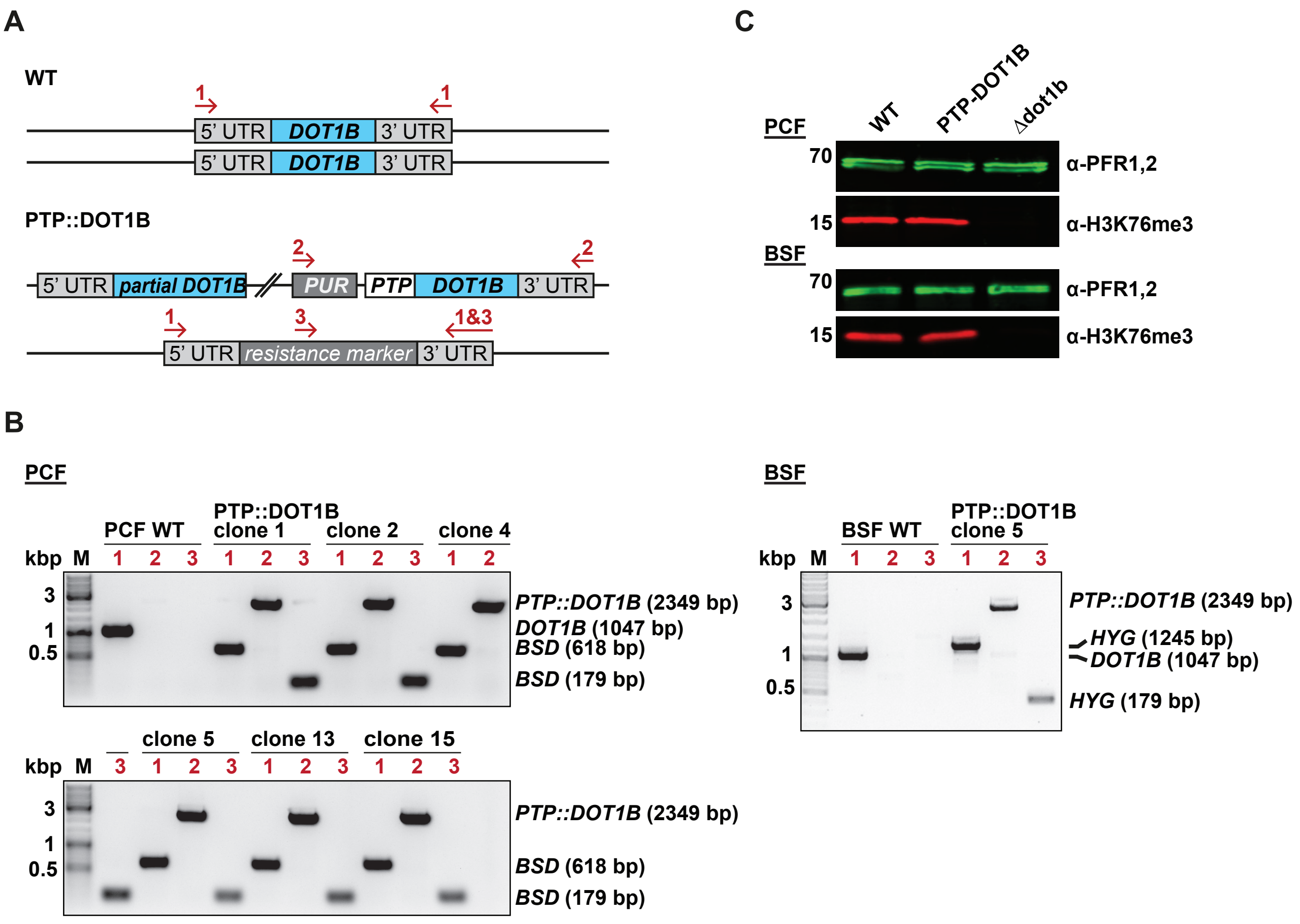

Supplementary Figure S1. PTP tagging of DOT1B in PCF and BSF trypanosomes. (**A**) Illustration of the endogenous *DOT1B* locus in WT and PTP::DOT1B cells. The *PTP* tag was fused to the 5' end of the first allele of *DOT1B* under puromycin (PUR) selection. The second allele of *DOT1B* was replaced by the blasticidin (*BSD*) resistance marker in PCFs, and by the hygromycin (*HYG*) resistance marker in BSF trypanosomes. Red arrows indicate the primers used for integration control by PCR. (**B**) Integration PCR with primers binding in the 5' and 3'UTR of *DOT1B* and within the resistance marker ORFs as indicated in A. Genomic DNA of six different clones were tested in PCF, one in BSF. Genomic DNA of WT cells was used as a control. Further studies in PCF were carried out with PTP::DOT1B clone 15. (**C**) Confirmation of the H3K76 trimethylation activity of the PTP-tagged DOT1B. Whole cell lysates of WT, PTP::DOT1B and Δdot1b cells were analyzed by immunoblotting with anti-H3K76me3 antibody. As a protein loading control, the same blot was probed with anti-PFR1,2 antibody.
