# Supplementary material for: A DOT1B/Ribonuclease H2 Protein Complex Is Involved in R-Loop Processing, Genomic Integrity, and Antigenic Variation in Trypanosoma brucei
Source: mBio. 2021 Nov 9;12(6):e01352-21. doi: 10.1128/mBio.01352-21 (PMC8576533; doi:10.1128/mBio.01352-21)
Supplement: FIG S2 [file mbio.01352-21-sf002.pdf]

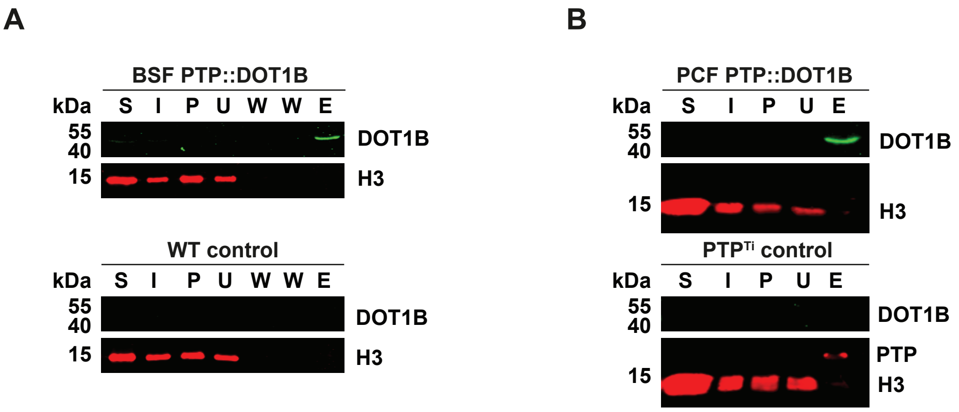

Supplementary Figure S2. Enrichment of DOT1B and the PTP^{Ti} control after affinity purifications. Representative Western blots with samples taken during the purification procedure of (**A**) PTP::DOT1B (49.5 kDa) and the WT control in BSF or (**B**) PTP::DOT1B and PTP^{Ti} control (18.6 kDa) in PCF. Whole cell lysates (S) were separated by centrifugation into soluble supernatants (I) and insoluble pellets (P). Supernatants were incubated with protein G sepharose beads. Further samples of unbound material (U), the subsequent washing steps of the beads (W), and of the proteins eluted from the beads (E) were taken. 16.5-fold more of the eluate was loaded compared to the other samples isolated during the purification procedure for the PCF pulldown and 15-fold more for the pulldown in BSF parasites. Blots were probed with anti-DOT1B antibody and anti-H3 antibody.
