# Supplementary material for: A DOT1B/Ribonuclease H2 Protein Complex Is Involved in R-Loop Processing, Genomic Integrity, and Antigenic Variation in Trypanosoma brucei
Source: mBio. 2021 Nov 9;12(6):e01352-21. doi: 10.1128/mBio.01352-21 (PMC8576533; doi:10.1128/mBio.01352-21)
Supplement: FIG S3 [file mbio.01352-21-sf003.pdf]

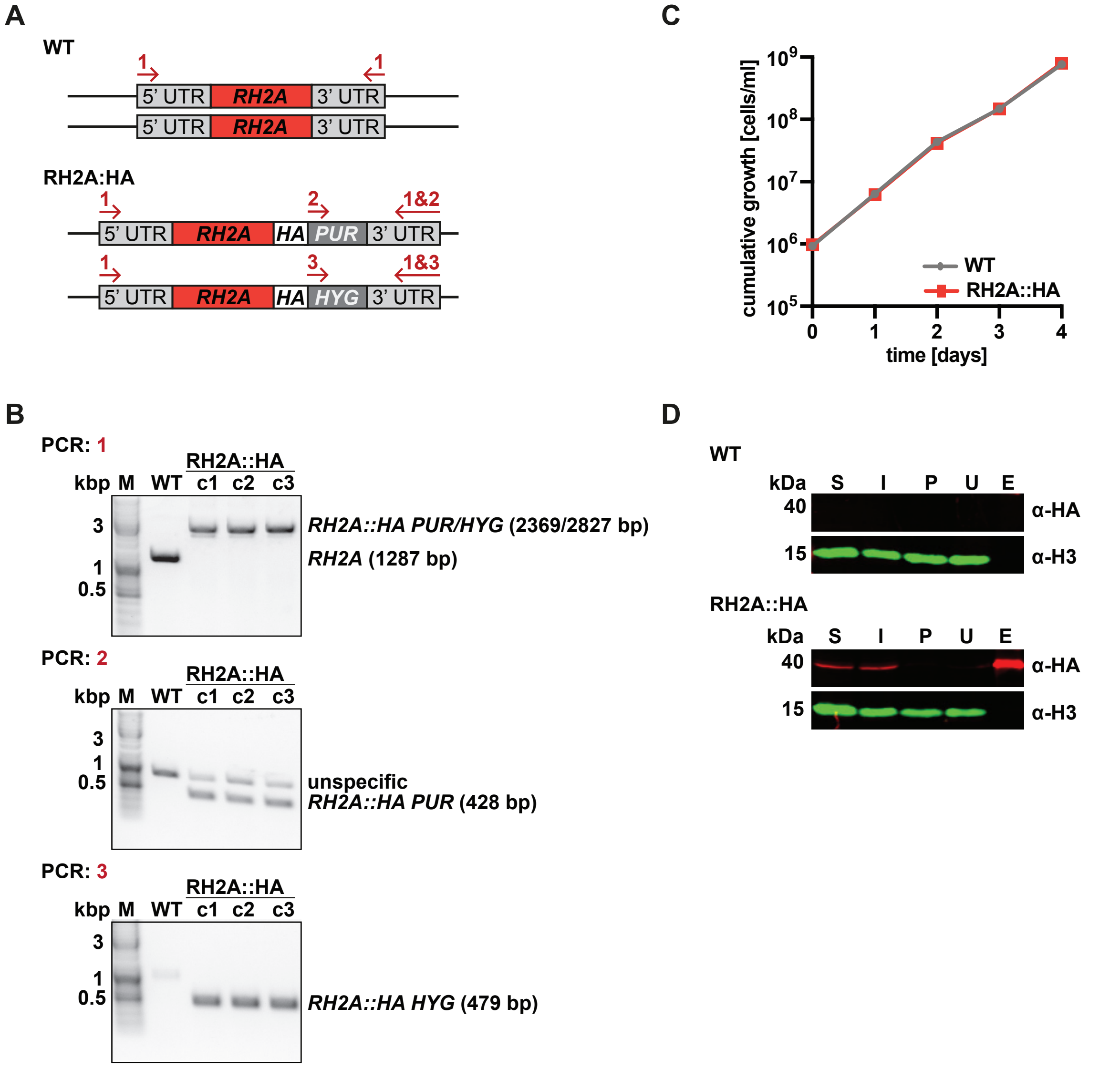

Supplementary Figure S3. HA tagging of *RH2A* in PCF trypanosomes. (**A**) Illustration of the *RH2A* gene locus in WT and RH2A::HA cells. The HA tag was fused to the 3' end of both alleles of *RH2A* in PCF trypanosomes. Arrows indicate the primers used for integration control by PCR. (**B**) Integration PCR with primers binding in the 5' and 3'UTR of *RH2A* and within the resistance marker ORFs, as indicated in A, verified integration of constructs. Genomic DNA of three different RH2A::HA clones was tested and genomic DNA of WT cells served as control. Further analysis in this study was carried out with RH2A::HA clone 1. (**C**) Cumulative growth shows no difference between PCF WT and RH2A::HA cells (n=3). (**D**) Representative WB of the RH2A-HA (39.3 kDa) and WT control IPs. Whole cell lysates (S) were separated by centrifugation into soluble supernatants (I) and insoluble pellets (P). Supernatants were incubated with anti-HA antibody conjugated to sepharose. Samples of unbound material (U) and of the eluates (E) were taken. 26-fold more of the eluate was loaded compared to the other samples isolated during the purification procedure. The average amount of purified RH2A-HA of the four biological replicates compared to the input material was 27.5%. The blots were probed with anti-HA and anti-H3 antibody.
