# Supplementary material for: A DOT1B/Ribonuclease H2 Protein Complex Is Involved in R-Loop Processing, Genomic Integrity, and Antigenic Variation in Trypanosoma brucei
Source: mBio. 2021 Nov 9;12(6):e01352-21. doi: 10.1128/mBio.01352-21 (PMC8576533; doi:10.1128/mBio.01352-21)
Supplement: FIG S4 [file mbio.01352-21-sf004.pdf]

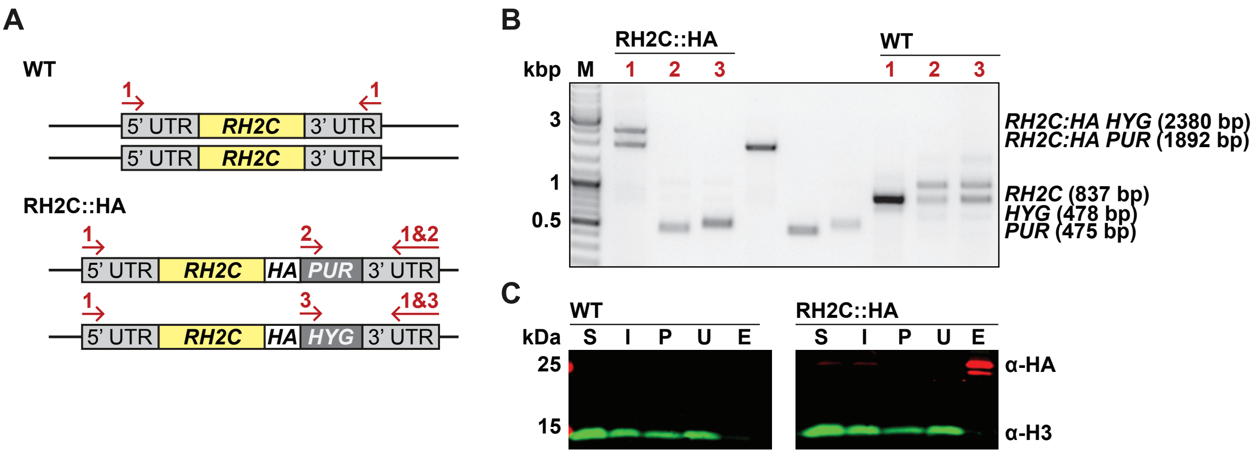

Supplementary Figure S4. HA tagging of *RH2C* in PCF trypanosomes. (**A**) Illustration of the *RH2C* gene locus in WT and RH2C::HA cells. The HA tag was endogenously fused to the 3' end of both alleles of *RH2C*. Arrows indicate the primers used for integration control. (**B**) Integration PCR with different primer combinations. Primers binding in the 5' and 3'UTR of *RH2C* were used to confirm the fusion of the HA tag to *RH2C*. Primers binding in the resistance marker ORFs and 3'UTR of *RH2C* confirmed 3' fusion of the tag. Genomic DNA of WT cells served as a control. (**C**) Representative WB with samples taken during the purification procedure of RH2C-HA (20.4 kDa). Whole cell lysates (S) were separated by centrifugation into soluble supernatants (I) and insoluble pellets (P). Supernatants were incubated with anti-HA antibody sepharose conjugates and samples of unbound fractions (U) and of the eluate (E) were taken. 21-fold more of the eluate was loaded compared to the other samples isolated during the purification procedure. Average RH2C-HA IP efficiency of quadruplicates was 25%. Samples were immunoblotted using anti-HA antibody and anti-H3 antibody.
