# Supplementary material for: A DOT1B/Ribonuclease H2 Protein Complex Is Involved in R-Loop Processing, Genomic Integrity, and Antigenic Variation in Trypanosoma brucei
Source: mBio. 2021 Nov 9;12(6):e01352-21. doi: 10.1128/mBio.01352-21 (PMC8576533; doi:10.1128/mBio.01352-21)
Supplement: FIG S5 [file mbio.01352-21-sf005.pdf]

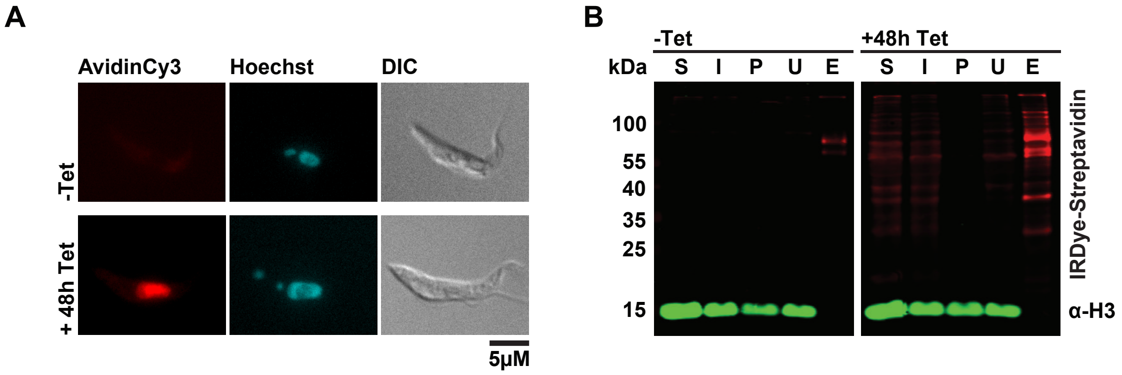

Supplementary Figure S5. Biotinylation of neighboring proteins by DOT1B-BirA*. (**A**) Immunofluorescence analysis of cells after incubation with biotin either with or without ectopic DOT1B-BirA* expression by addition of tetracycline (Tet). Biotinylated proteins labeled with fluorescently-conjugated avidin were observed in the nucleus. DNA was stained with Hoechst. (**B**) Representative WB with samples taken during the purification procedure after incubation with biotin of ectopically expressing DOT1B-BirA* cells or uninduced control cells. Whole cell lysates (S) were separated by centrifugation into soluble supernatants (I) and insoluble pellets (P). Supernatants were incubated with Streptavidin-conjugated agarose beads and samples of unbound fractions (U) and of the eluate (E) were taken. 38-fold more of the eluate was loaded compared to the other samples isolated during the purification procedure. Average purification efficiency of biotinylated proteins, calculated from the dominant 35 kDa protein from four replicate experiments, was 22.5%. Samples were immunoblotted using anti-H3 antibody and IRDye-Streptavidin.
