# Supplementary material for: A DOT1B/Ribonuclease H2 Protein Complex Is Involved in R-Loop Processing, Genomic Integrity, and Antigenic Variation in Trypanosoma brucei
Source: mBio. 2021 Nov 9;12(6):e01352-21. doi: 10.1128/mBio.01352-21 (PMC8576533; doi:10.1128/mBio.01352-21)
Supplement: FIG S6 [file mbio.01352-21-sf006.pdf]

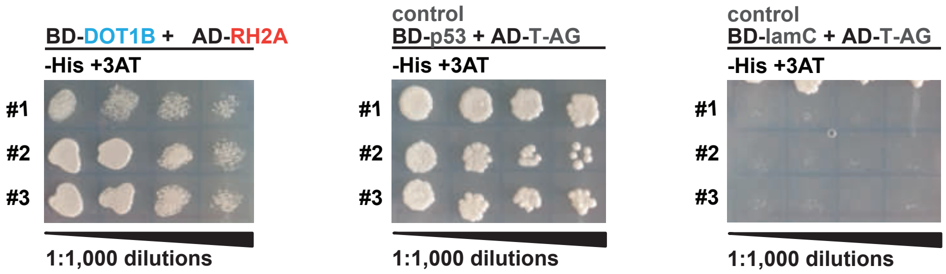

Supplementary Figure S6. Interaction between DOT1B and RH2A in the yeast 2-hybrid assay. Proteins of interest were either fused to the DNA-binding domain (BD) or to the activation domain (AD) of the yeast Gal4 transcription factor and were used to transform yeast cells. Growth of yeast in medium lacking histidine (-His) indicates interaction between the two proteins of interest. To reduce false positives, the stringency was adapted by growing cells in medium containing 2 mM 3-amino-1,2,4-triazole (3AT). Additionally, controls recommended by the manufacturer were probed.
