# Supplementary material for: A DOT1B/Ribonuclease H2 Protein Complex Is Involved in R-Loop Processing, Genomic Integrity, and Antigenic Variation in Trypanosoma brucei
Source: mBio. 2021 Nov 9;12(6):e01352-21. doi: 10.1128/mBio.01352-21 (PMC8576533; doi:10.1128/mBio.01352-21)
Supplement: FIG S7 [file mbio.01352-21-sf007.pdf]

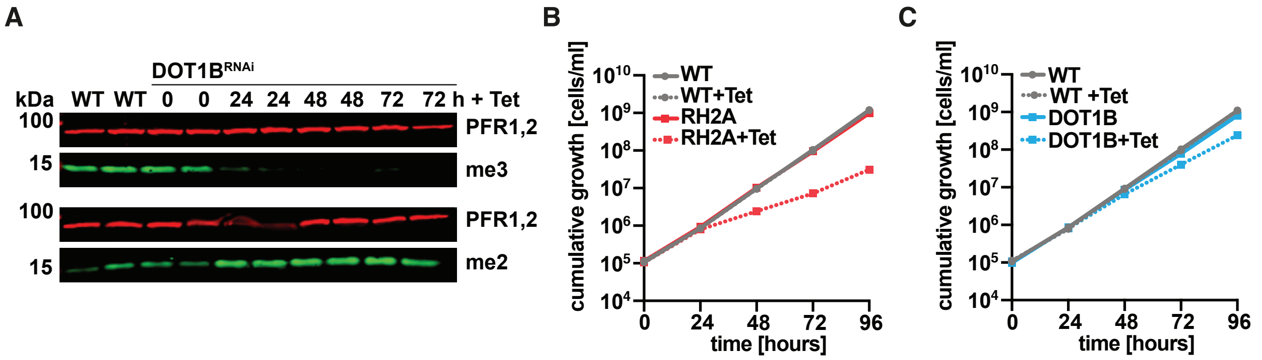

Supplementary Figure S7. Depletion of DOT1B and RH2A in *T. brucei* BSF. (**A**) WB with whole cell lysates taken during different timepoints after RNAi induction by addition of tetracycline (Tet), analyzed in duplicates. Decrease of DOT1B-specific H3K76me3 signal (me3) and the associated increase of H3K76me2 (me2) signal confirmed DOT1B depletion. Anti-PFR1,2 antibody was used as a loading control. (**B**) Depletion of RH2A using RNAi results in a strong growth defect. (**C**) Depletion of DOT1B using RNAi results in a mild growth defect (n=3).
