# Supplementary material for: A DOT1B/Ribonuclease H2 Protein Complex Is Involved in R-Loop Processing, Genomic Integrity, and Antigenic Variation in Trypanosoma brucei
Source: mBio. 2021 Nov 9;12(6):e01352-21. doi: 10.1128/mBio.01352-21 (PMC8576533; doi:10.1128/mBio.01352-21)
Supplement: FIG S8 [file mbio.01352-21-sf008.pdf]

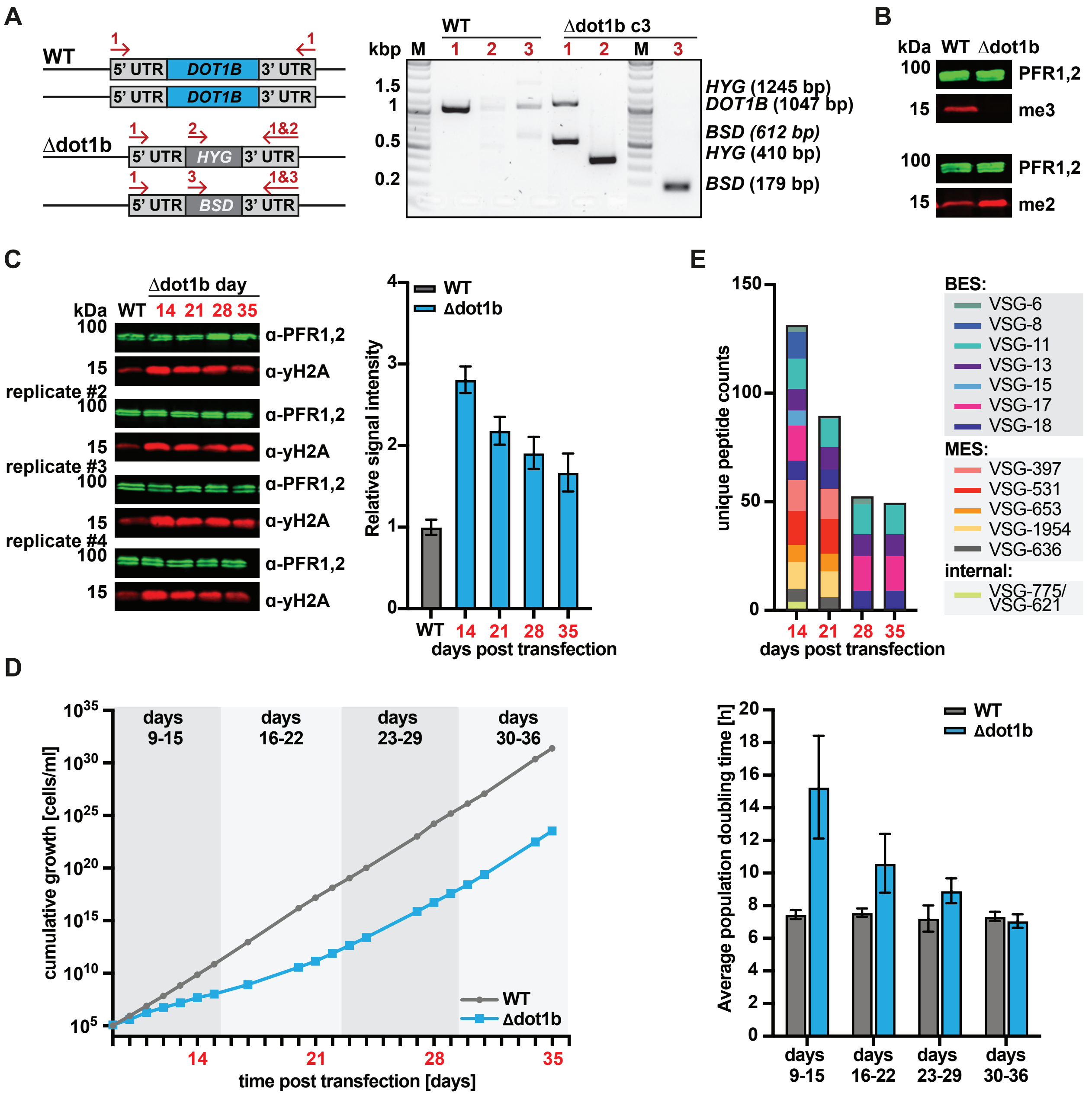

Supplementary Figure S8. Adaptation phenotypes of Δdot1b cells. (**A**) Illustration of the *DOT1B* gene locus in WT and Δdot1b cells. Alleles were replaced with resistance marker ORFs of hygromycin (*HYG*) and blasticidin (*BSD*). Arrows indicate the primers used for integration control. Primers binding in the 5' and 3'UTR were used to confirm the KO of *DOT1B*. Primers binding in the resistance marker ORFs and 3'UTR of *DOT1B* confirmed integration of respective markers at the right locus. Genomic DNA of WT cells served as a control M, marker lane. (**B**) Confirmation of the loss of H3K76me3 in Δdot1b cells with a corresponding increase of H3K76me2 signal. Whole cell lysates of WT and Δdot1b cells were analyzed by immunoblotting with anti-H3K76me3 and anti-H3K76me2 antibodies. As a protein loading control, the same blot was probed with anti-PFR1,2 antibodies. (**C**) WB and its quantitative analysis of DNA damage marker γH2A in Δdot1b cells 14, 21, 28 and 35 days post deletion of DOT1B. A reduction of DNA damage was observed over time. γH2A levels were normalized to PFR1,2 protein expression. WT level was set to 1. Error bars represent the standard deviation of the four biological replicates. (**D**) The severe growth defect of Δdot1b cells was gradually lost over a period of 5 weeks after generation of the KO cell line (n=4). The average population doubling time decreased over the same time period until it returned to roughly WT levels. (**E**) Mass spectrometry analysis of whole cell lysates reveals significant enrichment of multiple VSGs in Δdot1b compared to WT cells. BES-associated VSGs, MES-associated VSGs as well as VSGs from internal genome loci were deregulated during the early stages after KO generation; later only BES-associated VSGs were enriched.
