# Supplementary material for: A DOT1B/Ribonuclease H2 Protein Complex Is Involved in R-Loop Processing, Genomic Integrity, and Antigenic Variation in Trypanosoma brucei
Source: mBio. 2021 Nov 9;12(6):e01352-21. doi: 10.1128/mBio.01352-21 (PMC8576533; doi:10.1128/mBio.01352-21)
Supplement: FIG S9 [file mbio.01352-21-sf009.pdf]

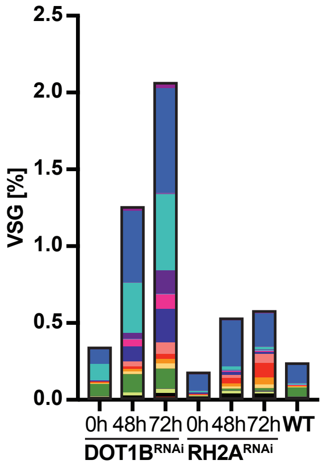

	Localization in ES:	Localization in genome:	fold enrichment of 72h DOT1B RNAi compared to WT:	fold enrichment of 72h DOT1B RNAi compared to 0h:	fold enrichment of 72h RH2A RNAi compared to WT:	fold enrichment of 72h RH2A RNAi compared to 0h:
VSG-3	BES 7	MBC 6	24.2	15.1	6.2	3.7
VSG-8	BES 12 / BES 14	MBC 2 / MBC 7	5.7	7.4	1.8	2.1
VSG-9	BES 2	IC	3.9	3.1	-	-
VSG-11	BES 15	MBC 3	66.2	4.8	2.1	1.3
VSG-13	BES 17	IC	120.5	66.7	2.5	1.1
VSG-15	BES 10	IC	2.1	2.1	5	4.7
VSG-17	BES 13	IC	68.2	42	4.4	3.7
VSG-18	BES 5	MBC 5	168.1	55.9	12.4	7
VSG-397	MES	MBC 10	56.9	38.8	43.5	31.3
VSG-531	MES	MBC 3	7.7	7.4	22	17.6
VSG-653	MES	MBC 11	5.6	4.7	9.7	35.3
VSG-1954	MES	MBC 8	16.3	20.9	9.8	12.2
VSG-636	MES	MBC 9	4.5	3.1	6.8	4.2
VSG-3591	MES	MBC 10	18.2	11.6	4	3.1
VSG-775	-	MBC 10	17.4	14.5	9	7.3
VSG-1828	-	MBC 9	1.5	1.4	1.9	1.6
VSG-3233	-	MBC 7	-	-	3.1	3.2
VSG-322	-	UD	2	1.1	5.1	3.4
VSG-336	-	MBC 8	2.2	1.6	-	5

Supplementary Figure S9. Increased expression of several VSGs after DOT1B or RH2A depletion. The graph shows the percentages of VSGs identified by mass spectrometry during the different timepoints after RNAi induction of DOT1B and RH2A cell lines. The parental cell line was analyzed as a control. The table displays the genomic localization of analyzed VSGs, showing that VSGs were deregulated from throughout the genome repertoire. In addition, the fold increase of each VSG value is shown 72 hours after RNAi induction compared to the values of uninduced and WT control. Interestingly, nearly the same VSGs were deregulated in the two different cell lines. BES (BSF expression site), MES (Metacyclic expression site), MBC (megabase chromosome), IC (intermediate chromosome), UD (undefined).
